# Exploring the Role of Artificial Intelligence in Evidence Synthesis: Insights From the CORE Information Retrieval Forum 2025

**DOI:** 10.1002/cesm.70049

**Published:** 2025-09-07

**Authors:** Claire H. Eastaugh, Madeleine Still, Fiona R. Beyer, Sheila A. Wallace, Hannah O'Keefe

**Affiliations:** ^1^ NIHR Innovation Observatory Newcastle University Newcastle‐upon‐Tyne UK; ^2^ Population Health Sciences Institute, Faculty of Medical Sciences Newcastle University Newcastle‐upon‐Tyne UK

**Keywords:** artificial intelligence, evidence synthesis, generative AI, information retrieval, information specialist, large language models, literature search

## Abstract

**Introduction:**

Information retrieval is essential for evidence synthesis, but developing search strategies can be labor‐intensive and time‐consuming. Automating these processes would be of benefit and interest, though it is unclear if Information Specialists (IS) are willing to adopt artificial intelligence (AI) methodologies or how they currently use them. In January 2025, the NIHR Innovation Observatory and NIHR Methodology Incubator for Applied Health and Care Research co‐sponsored the inaugural CORE Information Retrieval Forum, where attendees discussed AI's role in information retrieval.

**Methods:**

The CORE Information Retrieval Forum hosted a Knowledge Café. Participation was voluntary, and attendees could choose one of six event‐themed discussion tables including AI. To support each discussion, a QR code linking to a virtual collaboration tool (Padlet; padlet.com) and a poster in the exhibition space were available throughout the day for attendee contributions.

**Results:**

The CORE Information Retrieval Forum was attended by 131 IS from nine different types of organizations, with most from the UK and ten countries represented overall. Among the six discussion points available in the Knowledge Café, the AI table was the most popular, receiving the highest number of contributions (*n* = 49). Following the Forum, contributions to the AI topic were categorized into four themes: critical perception (*n* = 21), current uses (*n* = 19), specific tools (*n* = 2), and training wants/needs (*n* = 7).

**Conclusions:**

While there are critical perspectives on the integration of AI in the IS space, this is not due to a reluctance to adapt and adopt but from a need for structure, education, training, ethical guidance, and systems to support the responsible use and transparency of AI. There is interest in automating repetitive and time‐consuming tasks, but attendees reported a lack of appropriate supporting tools. More work is required to identify the suitability of currently available tools and their potential to complement the work conducted by IS.

## 1 Introduction

Artificial intelligence (AI) is an emerging technology with rapid advancements and increasing impact across various industries. Whilst methods such as natural language processing and predictive modeling have been available for many years, the emergence of generative AI (GenAI) has brought new promise to automation methodologies [1, 2], with the potential for increased accuracy and advanced data synthesis. This is timely as the pressures on researchers to conduct faster, more immediate evidence syntheses and produce outputs are growing [3, 4]. These pressures have arisen from a need to respond rapidly to emerging crises and policy issues. In the UK, there are substantial burdens on the National Health Service (NHS) which require prompt evidence‐driven action to mitigate, such as the substantial funding shortage, which could, in part, be addressed through evidence synthesis and economic evaluations of current practices [5, 6]. The COVID‐19 crisis demonstrated how rapid‐fire evidence can be used to inform practice and produce positive outcomes [7, 8]. However, the discussion regarding AI automation for systematic reviews has been ongoing for two decades [9, 10]. Tools to aid evidence synthesis are abundant and particularly well‐developed for screening, yet very little in the way for search tools. The joint call for papers on AI in evidence synthesis between Collaboration for Environmental Evidence (CEE), Campbell and Cochrane highlights this, and aims to bridge the gap between theory and practice [11].

Information specialist (IS) methodologies underpin all evidence synthesis efforts [12]. Without an expertly built search strategy the synthesis may be poor and the results likely biased [13]. Search strategy development is labor intensive and time consuming [14], often requiring several weeks from the initial scoping to finalized search [15, 16, 17]. IS are expert methodologists but are not necessarily subject specialists, meaning they are reliant on the wider team providing exhaustive terminology including lesser used phrasing and colloquialisms.

Furthermore, labor and time resources needed to manually curate the search strategies can easily spiral when multiple databases are used each database has its own syntax, search rules and subject headings which mean translating searches is notoriously difficult and requires cross validation of subject headings amongst the search strategies. Bullers et al. [16] reported that survey respondents took an average of 5.4 h to translate search strategies, but this task could take up to 75 h and was the most time intensive task for IS after designing the initial search strategy. The complexity and time required to conduct the search also varies by the intended use of the information, for example, searching for systematic reviews, grant proposals, or articles [18, 19]. Automating search strategy development and translation across databases is widely sought after [19], yet it has been historically difficult to develop accurate software to support this, notably due to the differences in ontologies used for subject headings [20, 21]. GenAI methodologies have potential to provide solutions, although there are potential challenges to effective AI solutions, including user confidence and skills in using these tools but also the paradigm used to develop such tools.

Core principles of traditional information retrieval and evidence synthesis such as transparency, reproducibility, precision, and the process of querying structured databases, are being challenged by GenAI and large language models (LLMs) [22]. These tools predict language patterns from vast, unstructured datasets and are increasingly synthesizing rather than retrieving which raises concerns about the reliability, transparency, and veracity, often referred to as “black box” [23, 24].

The professional development, recruitment, and retention of IS are increasingly shaped by the evolving demands of research synthesis and evidence‐based practice. The complexity and diversity of review methodologies require IS to possess both methodological expertise and adaptability [25, 26]. Meanwhile, emerging technologies are transforming traditional workflows, underscoring the need for ongoing training and support [27, 28].

In the UK, the National Institute of Health and Care research (NIHR) is the leading funder of health and social care research, with over £601 million spent on research funding, training and career development in 2023/24 [29]. It is closely aligned to the NHS through the Government's Department of Health and Social Care (DHSC). Although NIHR works as “one NIHR” it has a vast infrastructure. The NIHR Applied Health and Care Research Methodology Incubator was established in April 2020 with the aim of supporting research methodologists working in applied health and care research by raising awareness of research methodology careers and increasing methodology research capacity.

The NIHR Innovation Observatory is one of the NIHRs innovation hubs, specializing in horizon scanning and evidence synthesis. The NIHR Innovation Observatory, supported by a seedcorn grant from the NIHR Methodology Incubator, undertook research in 2023 to identify barriers and facilitators to career progression and research for IS, and solutions to overcoming these barriers [30, 31]. Due to the wide variety of settings and sectors, roles, specializations and job titles [32], the project team described a range of tasks and skills under the umbrella term IS which will be adopted here. The definition of IS used throughout the project was:

“…anyone, regardless of job title or qualifications, who works, or has worked in a role in which they are undertaking activities such as literature searching, information retrieval, reference management, writing for publication and teaching about these topics, or who is undertaking methodological research in this area. These tasks could be carried out as part of a (clinical/social care) project team or could be related to your own information‐focused research. We also include in our definition people who have worked/qualified in IS fields and have transitioned to (other) methodologist/reviewer roles in teaching or research teams” [30, 31].

The final report found there was a demand for IS to build a community of practice, and for a central hub for collaboration, networking, and resource sharing [30, 31]. In response to these results, NIHR Innovation Observatory and NIHR Methodology Incubator co‐sponsored the inaugural CORE Information Retrieval Forum (CORE) which took place in January 2025 [33].

The forum was launched to bring together IS, and people who specialize in information retrieval, systematic searching, knowledge or data mining, evidence synthesis, archiving or reference management. Following a team science collaborative approach, the forum welcomed IS from all disciplines with the aim to share knowledge and experience, and to connect with others in the information community and discuss innovations through four objectives: developing the Community, highlighting Opportunities, exploring Research, and sharing Experience (CORE).

As part of CORE a Knowledge Café [34] was held where, amongst the six pertinent topics to IS' daily practice and careers, we explored their views of AI with the intention to better understand the perception, current application and needs of AI for IS, considering older methodologies such as machine learning, and newer technologies such as GenAI [35]. Here, we detail the findings and discuss the implications for careers and IS methodologies.

## 2 Methods

The Knowledge Café [34] was held in the afternoon of the 1‐day event for an hour. Participation in the workshop was voluntary. Attendees were able to select which of the six discussions to contribute to and had the freedom to move to different discussions during the hour. Each table hosted a subject relevant to the themes of the forum and IS: AI; search sources; experience; networking; professional development; and keeping the momentum of the forum going. Each subject had thought‐provoking questions to assist thinking about the issue. The AI topic asked: How do you use AI? How do you want to use AI? What concerns do you have? How do we spot if any information we review/critique has been AI generated? We did not limit the type of AI or underpinning methodology of tools in the discussion, inviting thought across the breadth of AI.

Supporting the workshop data gathering was a QR code linking to a virtual collaboration tool (Padlet; padlet.com) and a poster in the exhibition space where attendees could hand‐write their responses, both available for the full duration of the forum and mentioned throughout the day. During the workshop, a member of the NIHR Innovation Observatory Information Team (HOK) facilitated the discussion of the AI table. This role was to encourage dialogue, ask probing questions, and promote using the QR code to capture participants thoughts. Attendees were not restricted in the number of contributions they could make and could submit comments anonymously via the poster or QR code.

Following CORE, grounded theory [36] was employed to analyze the contributions to the AI topic from across the workshop discussion, virtual collaboration tool, and posters. The contributions were coded line by line before being grouped together and core categories selected. This process was conducted by one reviewer (H.O.K.) and verified by a second (C.H.E.) and performed in Microsoft Excel. The themes identified were:

Critical perception

This theme considers the concerns, limitations, and ethical considerations surrounding the adoption and use of AI in the IS space. It includes critical thinking and appraisal of AI, limitations, and transparency of where AI is being used.

Current uses

This theme refers to the practical, real‐world applications of AI across the varied space IS inhabit. This includes productivity, decision‐making, and information management.

Specific tools

This theme encompasses the AI tools which are actively being used by IS.

Training wants/needs

This theme reflects areas where participants highlighted additional knowledge, skills, or support to effectively engage with AI and machine learning tools to effectively use AI within their professional space. This includes research, support, and teaching.

## 3 Results

**Figure 1:**
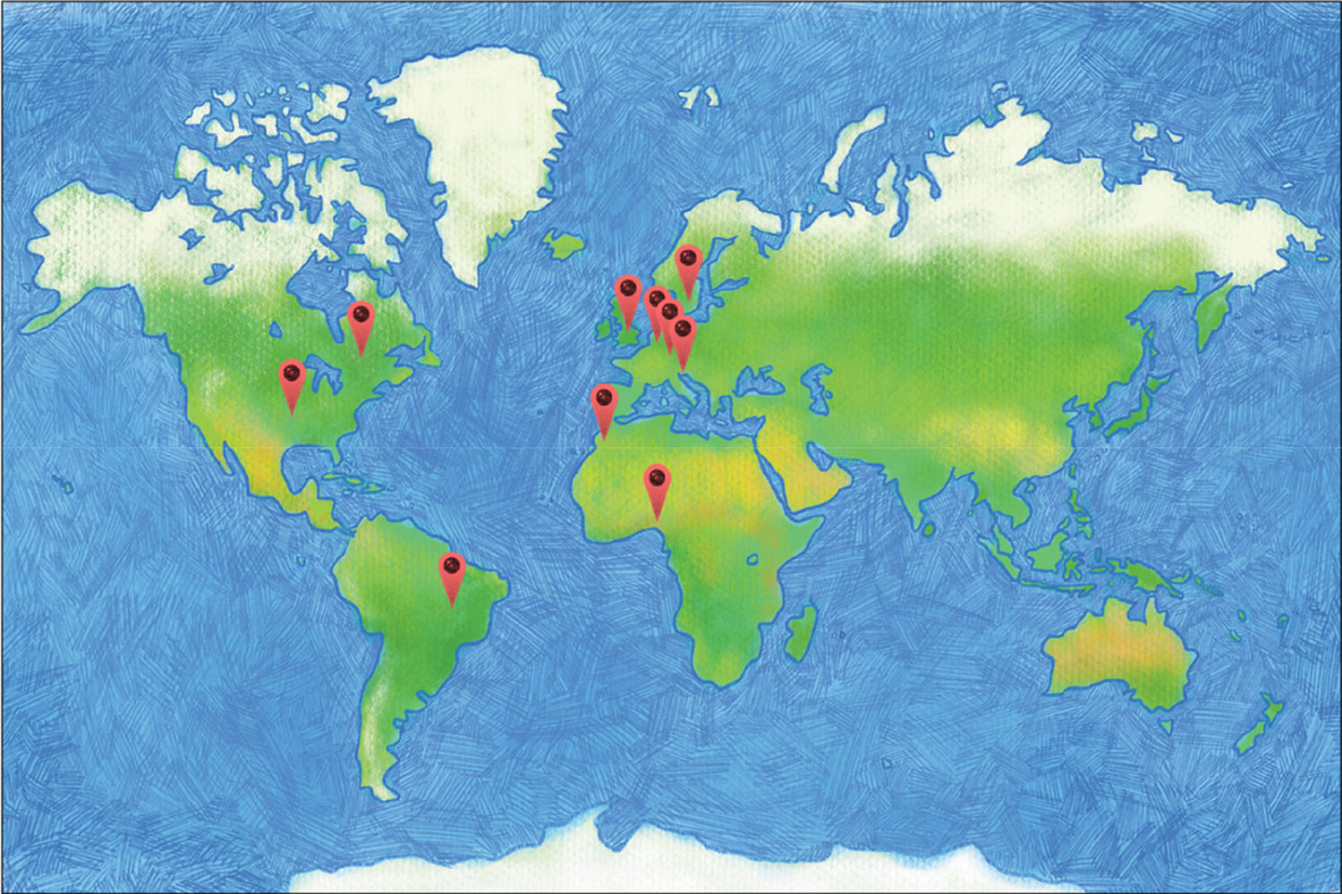
Global distribution of attendees to the CORE Information Retrieval Forum 2025. Map image by mtj6596 from Pixabay.

The CORE Information Retrieval Forum 2025 was attended by 131 IS from across nine different types of organizations, sectors, or roles affiliated with health and care, research, or academia (Table 1). These included Universities (*n* = 83), the NHS (*n* = 20), and National Institute for Health and Care Excellence (NICE) (*n* = 4). Most attendees were from across the United Kingdom (*n* = 119). It is unclear how many IS currently work in the UK, as existing figures include other information and library related roles [25]. However, anecdotally we understand that many IS work alone or in very small teams, so it seems reasonable to assume that this represents a considerable proportion of UK IS. CORE also attracted attendees from all over the world (Figure 1). This included the United States of America (*n* = 3), Slovakia (*n* = 2), and Brazil, Canada, Germany, Morocco, Netherlands, Nigeria, and Sweden (all *n* = 1).

**Table 1:** CORE Information Retrieval Forum 2025 attendees' distribution by sector.

Sector	No. of attendees
University	83
NHS	20
Industry	11
Research group	7
NICE	4
Government	2
NIHR	2
Freelance	1
PPI	1

Abbreviations: NHS = National Health Service, NICE = National Institute of Health and Care Excellence, NIHR = National Institute of Health and Care Research, PPI = Patient and Public Involvement.

### 3.1 Knowledge Café

Of the six discussion points available in the Knowledge Café, the AI table was the most subscribed with up to 16 participants at its high point, it also received the greatest number of total contributions (*n* = 49) (Table 2).

**Table 2:** Total contributions per topic.

Topic	No. of contributions^a^
Artificial intelligence (AI)	49
Keeping the momentum of the forum going	29
Experience	23
Networking	12
Search sources	11
Professional development	1

^a^ Number reflects individual contributions, some responses held multiple points.

Following CORE, contributions to the AI topic were categorized into four themes (Table 3), critical perception (*n* = 21), current uses (*n* = 19), specific tools (*n* = 2), and training wants/needs (*n* = 7).

**Table 3:** AI topic discussion breakdown of contributions against theme.

Theme	Contribution	No. of responses
Critical perception	Reliability, reproducibility, transparency, and bias of data ingested in LLMs	5
	Not ready/suitable for use cases	4
	Fast changing pace and lack of clear guidance	3
	Encourages taking shortcuts	3
	Lack of knowledge or understanding	2
	Avoiding use	1
	Impact on the environment	1
	Need for critical appraisal and critical thinking	1
	Screening—what point it is safe to stop	1
Current uses	Explore search terms, scoping	6
	Create presentations	2
	Sense‐check and advise	2
	Evidence summaries	2
	Create efficiencies where certainty is not needed	2
	Data visualization	2
	Supporting NHS staff to understand limitations and pitfalls	1
	Data extraction	1
	Finding synonyms for search strategies	1
Training wants/needs	Reduce manual screening/machine learning assisted screening	2
	Algorithms and supervised ML for identifying patterns	1
	Research and teaching	1
	Speed up boring tasks (efficiency)	1
	How to spot the use of AI in papers/reviews	1
	ML classifiers for record screening	1
Specific tools	Microsoft Power BI	1
	Google NotebookLM	1

Abbreviations: LLM = large language model, ML = machine learning.

A range of critical perspectives on the incorporation of AI tools in professional and research contexts, particularly LLMs, were expressed. Concerns surrounded the trustworthiness, transparency, and reproducibility of data, as well as inherent bias (*n* = 5). Underscoring this were reservations that AI tools are not yet fit‐for‐purpose in information‐related work (*n* = 4) and that as AI is rapidly evolving, there is insufficient guidance or standards for its application (*n* = 3). Furthermore, there were concerns that AI promotes taking risky shortcuts (*n* = 3) which could reduce the likelihood of human input to critically think and critically appraise the outputs (*n* = 1). There was also acknowledgment that some IS have a lack of understanding or expertise in AI (*n* = 2). One attendee noted they avoided using AI at all (*n* = 1) but did not specify why. Other issues include the environmental impact (*n* = 1); the infrastructure required to support AI produces electronic waste at the data centers housing AI servers, and there is a reliance on critical minerals and rare earth elements in the processing, storing, and transmission of information, and significant water consumption through cooling systems [37]. Another attendee noted the issue of AI predicting when to stop screening search results for eligibility (*n* = 1). Although several stopping rules have been proposed, effectiveness has not been robustly evaluated [38].

Participants highlighted a variety of practical applications where AI tools already support their work within the IS space. Most common was using AI to explore keywords and search terms, and scoping topics (*n* = 6), with specific reference to finding synonyms (*n* = 1). Evenly split were instances where participants reported using AI for efficiency gains (*n* = 2), creating content through evidence summaries (*n* = 2), presentations (*n* = 2), and visualizations (*n* = 2). Other points include data extraction (*n* = 1) and supporting NHS staff to understand limitations and pitfalls (*n* = 1) though the participant did not expand on what these were.

Participants only explicitly noted they currently use Microsoft Power BI (*n* = 1) and Google NotebookLM (*n* = 1). Both incorporate GenAI features using Copilot and Gemini. The participants did not expand on how they are used, but potential use cases include visualization of search terms and linkage, summarizing papers and identifying keywords.

Finally, there was a curiosity toward for AI in research and teaching (*n* = 1) with participants highlighting an interest in using AI and machine learning for efficiency purposes (*n* = 1) with an emphasis on reducing manual screening (*n* = 2) and classifiers for record screening (*n* = 1). Pattern recognition from machine learning (*n* = 1) and identifying AI generated research outputs (*n* = 1) are also important.

## 4 Discussion

The CORE Knowledge Café aimed to explore the views of IS on AI with the intention to better understand the perception of AI, the role AI will play within the IS space, the current application of AI, and any hesitancy towards the adoption of AI. The views expressed by the attendees were categorized into four main themes: critical perception, current uses, specific tools and training wants/needs. From these themes, four overall areas stand out for consideration.

### 4.1 Eagerness to Explore Current AI Tools

Participating IS reported they are increasingly exploring and using AI tools to support a range of tasks including scoping search terms and synonyms, creating presentations and visualization. These tools also assist with data extraction, producing evidence summaries while creating efficiencies in areas where high certainty is not essential. AI offers promising enhancements to information retrieval though Hersh reports that it should complement, not replace, traditional search systems and an ongoing need for research into how AI can be responsibly integrated [22]. Other research notes there may be efficiency gains; AI does not yet surpass expert‐led search strategies in accuracy or comprehensiveness, especially in contexts demanding methodological rigour [27, 39].

### 4.2 Hesitancy to Adopt

Whilst there has been little research in this area, anecdotally we know hesitancy to adopt AI does not come from concerns of job security and changes in workflow, but rather concerns about transparency, accuracy, reproducibility, and insufficient guidance or standards. The closing keynote speaker at CORE, Su Golder, discussed the progression of IS work, from handsearching physical paper records to floppy disks, to the revolution of the internet, concluding that there is little likelihood of AI replacing an IS [33].

Research that has taken place advocating AI to assist or complement the work conducted by an IS note the complexity and nuances of IS undertakings and the difficulties faced with accuracy and robustness [23, 39]. The contributions made to the AI discussion at CORE would support this, as concerns lay in the ethical questions around using AI including data privacy and bias in AI algorithms, as well as reservations on the quality of the data, low accuracy, illegitimate referencing, reproducibility of results, and ambiguous sourcing.

### 4.3 Training and Guidance

Whilst institutions such as Cochrane and the Chartered Institute of Library and Information Professionals (CILIP) offer training for IS, and AI‐related webinars are available, there remain limited specific training opportunities surrounding the practical application and embedding of AI for IS workflows. Instead, IS must seek training from computational science institutes which is often geared towards more technical applications. Interestingly, automation for screening was highlighted most within training needs, indicating that IS roles are expanding and evolving from search strategy development. Many semi‐automated screening tools are available with comprehensive guidance. This suggests that these tools and resources are not directed efficiently towards IS, most likely because screening is not seen as a traditional IS role. Given the diversity of IS careers, it may be that resources are not available under certain institutional cyber safety policies, contractual restrictions, prohibited use, licensing restrictions or that funding does not cover the licensing fees, and thus access is limited or unequal, even to IS within a single institution. This can result in unequal opportunities for research, create disparity in search precision and completeness depending on accessibility and licensing terms, and affect professional development, particularly in smaller organizations or under budget constraints, risking a digital divide within the information science community. In any case, there is not a one‐size‐fits‐all solution for training and development. Training must be tailored to different career routes and levels of computational proficiency.

Integrating AI into professional development is vital to ensure IS stay current with evolving research and knowledge practices [26, 27, 28]. Lack of AI literacy characterizes reluctance to implement: how does AI work and how can AI be effectively integrated into existing processes? Implementing AI may be resource intensive, requiring time, a steep learning curve, and investment in training. For AI tools to be optimally used, IS require their institutions to allow them capacity for training in this space. Staying current also requires continuous learning and adaptability, which can be difficult without institutional support and workflow demands placed on IS.

### 4.4 Balancing Efficiency and Critical Thinking

There is an evident desire to improve efficiency, particularly with monotonous or time‐consuming tasks, but concerns remain about the impact of this on the rigour and validity of the search process and the subsequent implications for the evidence base. It is important to acknowledge that IS are often part of a wider research team and careful deliberation and consensus is required to balance efficiency and comprehensiveness.

Guidance is in development to ensure the robust and transparent use of AI [40]. These guidelines highlight that responsible use should be everybody's concern, including developers, evidence synthesists, methodologists, funders, publishers and institutions. Viewing AI use as a collective concern and shared responsibility could help to minimize biases and increase the reliability of the outcomes. By improving the human oversight throughout the process, a fundamental level of critical thinking at each stage, from development to use in earnest, will be formed. The question remains whether this will be enough to endow trust and reassurance in the IS community, particularly when the results here suggest IS value critical thinking above efficiency. Contributions made to the Knowledge Café discussion indicated that IS currently see little or no benefit to using AI over traditional search methods beyond the idea of time saving and sense‐checking. Work is being conducted by several groups to explore retrieval platforms such as Lens.org, OpenAlex, and Elicit. Anecdotal evidence from webinars and professional groups suggests that AI tools like these do not yet improve on an expert IS search but may complement it [24].

### 4.5 Strengths and Limitations

This study has real‐world relevance as it addresses current and practical concerns of IS regarding AI integration and aligns with ongoing technological evolution and professional development needs within the IS field. The Knowledge Café format encouraged open discussion and anonymous contributions, fostering honest and diverse input, however contributions lack elaboration or specificity which constrained the analysis. As participation was voluntary, it possibly attracted those already interested in AI therefore creating potential bias in self‐selection. While contributions were encouraged via multiple media, it is acknowledged that this is a small sample and may not fully represent the broader IS community; however the participation at CORE and interest in AI within the IS community is encouraging for ongoing research and future development of the profession.

## 5 Conclusion

The views of IS who attended CORE suggest that though there are several critical perspectives on the integration of AI in IS practice and research contexts, it is not because of a reluctance to adapt and adopt. Rather, and it could be argued by their very nature, IS want more information. The results suggest a demand to automate or assist repetitive and time‐consuming tasks and for structure, education and training, ethical guidance, and systems in place to support the responsible use and transparency of AI. IS want a balance between automation and critical thought, to be well‐equipped to understand AI and be well placed to moderate AI's limitations. This can be achieved through targeted training of AI tools to IS and access to guidance currently in development for the transparent use of AI. As attendees of CORE reported a current scarcity of AI tools for information work, more work is required to identify current availability and the potential of these tools to complement the work conducted by IS. Efforts to target any currently available AI tools towards the IS profession are needed to provide IS with the information they need to feel competent in using these tools to assist in routine tasks. With the willingness to adapt and adopt, the diverse skill set, and demand to affect change, IS are most qualified to conduct and contribute to this study. The involvement of IS in research on AI tools geared to their own profession ensures that innovations are compatible and integrated with existing workflows and practice. This is essential to increase adoption and maximize the impact of AI in the IS community.

## Author Contributions

Claire Eastaugh and Hannah O'Keefe were responsible for conceptualization of the manuscript, data curation and for writing of the first version of the manuscript. Madeleine Still was responsible for checking the accuracy of the data analysis and adherence to the SQRQ reporting checklist. Fiona Beyer aided data curation and was responsible for supervision and project administration with Sheila Wallace. All authors were responsible for the review and editing of this manuscript for substantial intellectual content.

## Ethics Statement

The CORE Forum event and resulting outputs were deemed exempt from the need for ethical approval. Authors have followed the Standards for Reporting Qualitative Research (SRQR) checklist in the writing of this manuscript. A copy of the checklist is available in Supporting Information S1: Material 1.

## Conflicts of Interest

The authors declare no conflicts of interest.

## Peer Review

The peer review history for this article is available at https://www.webofscience.com/api/gateway/wos/peer-review/10.1002/cesm.70049.

## Supporting information

cesm70049‐sup‐0001‐CORE25_AI_SRQR_Checklist

## Data Availability

The data that support the findings of this study are available on request from the corresponding author. The data are not publicly available due to privacy or ethical restrictions.
